# Transcriptional Profile of *Mycobacterium tuberculosis* Replicating in Type II Alveolar Epithelial Cells

**DOI:** 10.1371/journal.pone.0123745

**Published:** 2015-04-06

**Authors:** Michelle B. Ryndak, Krishna K. Singh, Zhengyu Peng, Suman Laal

**Affiliations:** 1 Department of Pathology, New York University Langone Medical Center, New York, New York, United States of America; 2 Institutes of Biomedical Sciences, Shanghai Medical College, Fudan University, Shanghai, China; 3 Veterans Affairs New York Harbor Healthcare System, New York, New York, United States of America; Institut Pasteur, FRANCE

## Abstract

*Mycobacterium tuberculosis* (*M*. *tb*) infection is initiated by the few bacilli inhaled into the alveolus. Studies in lungs of aerosol-infected mice provided evidence for extensive replication of *M*. *tb* in non-migrating, non-antigen-presenting cells in the alveoli during the first 2–3 weeks post-infection. Alveoli are lined by type II and type I alveolar epithelial cells (AEC) which outnumber alveolar macrophages by several hundred-fold. *M*. *tb* DNA and viable *M*. *tb* have been demonstrated in AEC and other non-macrophage cells of the kidney, liver, and spleen in autopsied tissues from latently-infected subjects from TB-endemic regions indicating systemic bacterial dissemination during primary infection. *M*. *tb* have also been demonstrated to replicate rapidly in A549 cells (type II AEC line) and acquire increased invasiveness for endothelial cells. Together, these results suggest that AEC could provide an important niche for bacterial expansion and development of a phenotype that promotes dissemination during primary infection. In the current studies, we have compared the transcriptional profile of *M*. *tb* replicating intracellularly in A549 cells to that of *M*. *tb* replicating in laboratory broth, by microarray analysis. Genes significantly upregulated during intracellular residence were consistent with an active, replicative, metabolic, and aerobic state, as were genes for tryptophan synthesis and for increased virulence (ESAT-6, and ESAT-6-like genes, *esxH*, *esxJ*, *esxK*, *esxP*, and *esxW*). In contrast, significant downregulation of the DevR (DosR) regulon and several hypoxia-induced genes was observed. Stress response genes were either not differentially expressed or were downregulated with the exception of the heat shock response and those induced by low pH. The intra-type II AEC *M*. *tb* transcriptome strongly suggests that AEC could provide a safe haven in which *M*. *tb* can expand dramatically and disseminate from the lung prior to the elicitation of adaptive immune responses.

## Introduction

Upon inhalation into the alveolus, *Mycobacterium tuberculosis* (*M*. *tb*) is believed to be taken up by the alveolar macrophages. The mechanisms of intra-macrophage survival of *M*. *tb* have been studied extensively [1–3]. However, there are approximately 50 macrophages and 30,000 AEC per alveolus [4–6]. Evidence for *M*. *tb* infection of AEC in humans was initially demonstrated by detection of *M*. *tb* DNA in type II AEC in autopsied lung tissue of non-TB subjects from a TB-endemic region who had died of causes other than TB, i.e., in persons latently infected with *M*. *tb* [7]. Recent studies have demonstrated both *M*. *tb* DNA and viable bacteria in non-macrophage cells not only in the lungs but also in kidney from a TB-endemic region and spleen in latently infected subjects [8].

Studies of events that occur post-primary infection with *M*. *tb* cannot be done in humans, but studies in animal models provided evidence for bacterial replication in and dissemination from the lungs *prior* to elicitation of adaptive immunity [9–11]. Studies in aerosol-infected mice demonstrated extensive replication (>20,000 fold) of *M*. *tb* in a “non-migrating” compartment which does not present *M*. *tb* antigens to naïve CD4+ cells during the first 2–3 weeks post-infection [11]. Together these studies indicate that post-inhalation, besides macrophages, *M*. *tb* likely also invades and replicates in the AEC, a permissive environment for rapid replication, shielding from phagocytic cells, and possibly, acquisition of an invasive phenotype that facilitates bacterial dissemination to achieve systemic infection.

A549 is a human type II AEC carcinoma cell line used extensively for studies of asthma, lung injury/repair, toxic effects of particulate matter, COPD, effects of smoking etc [12–16]. Like primary type II AEC, A549 cells contain lamellar bodies that produce surfactant and have phospholipid content similar to that of *in situ* type II AEC [17]. A549 monolayers are polarized [18, 19] and therefore, correlate with type II physiological orientation. A549 cells are also being used to delineate the interactions of lung pathogens such as Influenza virus, *S*. *aureus*, *S*. *pneumonia*, etc within the AEC [20–22], and several investigators have studied *M*. *tb* infection of A549 cells [23–30]. Microscopic analysis locates *M*. *tb* to endocytic vacuoles in these cells [29], and A549 cells provide a permissive environment in which *M*. *tb* replicates >55-fold over a period of 7 days compared to 3-fold in human macrophages [31]. Both laboratory and clinical strains of *M*. *tb* invade and replicate in A549 cells, but only virulent strains are cytotoxic for them [24, 26, 29, 30, 32]. *M*. *tb* produces cell-wall and secreted proteins that promote *M*. *tb*:A549 interaction [32–34]. An *in vitro* bilayer alveolar model comprising of monolayers of A549 cells and EAhy926 cells (human endothelial line) grown on opposite sides of permeable membranes demonstrated migration of free *M*. *tb* and *M*. *tb*-laden macrophages across the alveolar barrier [25, 27]. Importantly, bacteria isolated from A549 cells after intracellular replication for 3 days demonstrated increased ability to traverse across the alveolar barrier model and to invade endothelial cells [27]. Moreover, BCG is attenuated for migration of the free bacteria across the alveolar barrier, and their passage through A549 cells does not enhance the invasive phenotype.

In the current study, we investigated the *M*. *tb* transcriptional adaptation of *M*. *tb* replicating in A549 cells in comparison to *M*. *tb* logarithmically grown in laboratory broth as the reference. The *M*. *tb* transcriptome during intracellular replication confirms that AEC provide a permissive environment for bacterial replication and transition to an invasive/disseminative phenotype. Importantly, the *M*. *tb* transcriptome in A549 cells is distinct from that reported during *M*. *tb* adaptation to the intra-macrophage environment. These results strongly suggest that *M*. *tb* could exploit AEC as a “safe haven” in which to expand and acquire a phenotype that enables rapid dissemination and seeding of the body during primary infection.

## Results and Discussion

### *M*. *tb* alters its transcriptional profile in type II AEC

**Fig 1:**
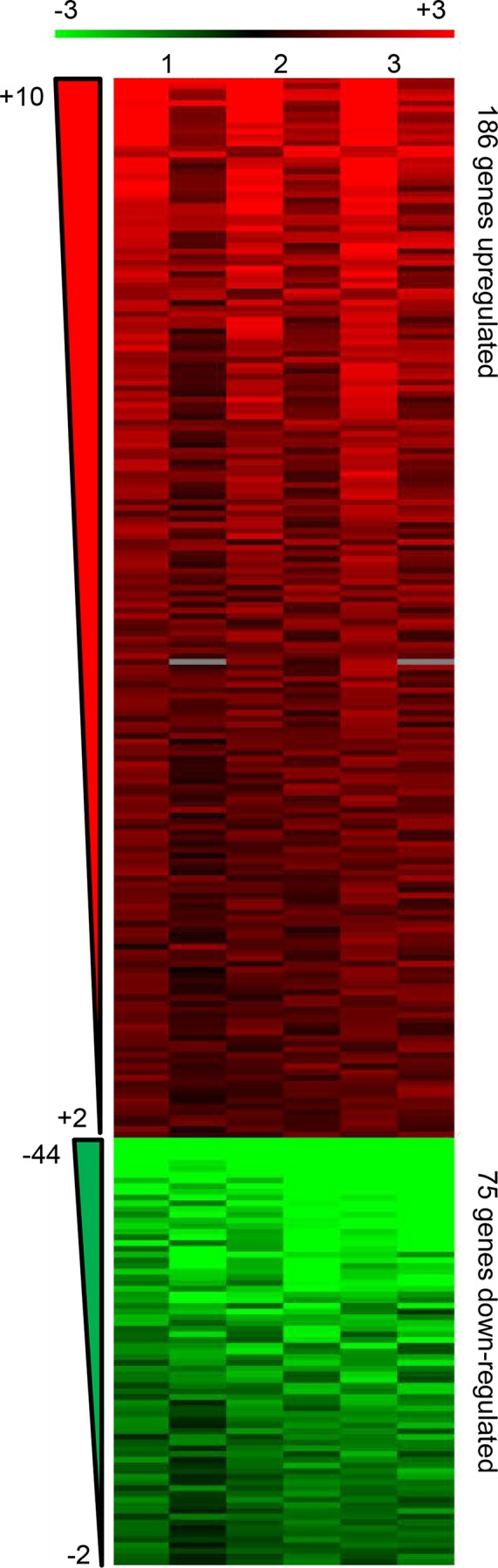
Heat map of *M*. *tb* genes differentially expressed at 72 hr replication in A549. Heat map denoting upregulated genes in red (186) and down-regulated genes in green (75) in *M*. *tb* H37Rv replicating in A549 compared to *M*. *tb* H37Rv growing logarithmically in 7H9 broth as the reference. Shown are the results from 2 biological replicates (Lanes 1 and 2- one biological replicate, four technical replicates including two dye flips; Lane 3- one biological replicate, two technical replicates including one dye flip) in order of degree of fold change from most differentially expressed to the cut-off (+2 for upregulated or -2 for downregulated). Ranges of fold change are indicated to the left.

To define the transcriptional profile of *M*. *tb* during infection of type II AEC, the post-infection (pi) time point of 72 hr (3 days) was chosen because earlier studies have demonstrated that *M*. *tb* is located in endosomes of A549 cells at this time [29]. Moreover, *M*. tb cultured in A549 cells is cytotoxic to these cells by days 4–5 pi [23]. Time points prior to day 3 would not provide adequate amounts of bacteria required to carry out the microarray experiments described in this study. At 72 hr of replication inside type II AEC, 261 *M*. *tb* genes were differentially expressed compared to *M*. *tb* grown in 7H9 laboratory broth (Fig 1). Of these, 186 genes were upregulated and 75 downregulated with fold changes ranging from +2 to +10 and -2 to -44 fold, respectively (Fig 1). The degree of correlation amongst replicates (biological and technical) is evident in the heat map (Fig 1), and the effects of dye flip are similar across the three experiments.

Numbers of differentially expressed genes were sorted by functional category as designated in the Tuberculist website (http://tuberculist.epfl.ch) (Table 1). By raw numbers, the most affected categories were “Intermediate metabolism and respiration” (68), “Cell wall and cell processes” (52), and “Conserved hypotheticals” (46). By percentage of representation in the *M*. *tb* genome, the most affected functional categories were “Information pathway” (11.2%), “virulence, detoxification, and adaptation” (9.6%), and “lipid metabolism” (8.5%). By number, the most upregulated categories were “Intermediate metabolism and respiration” (59), “Cell wall and cell processes” (38), and “Information pathways” (25) together accounting for two thirds of the upregulated genes, indicating an active metabolic and replicative state. The category “Conserved hypotheticals” solely accounted for more than one third (28) of the downregulated genes. Interestingly, of these 28 downregulated genes, nearly half (13) are upregulated (and the remainder not differentially expressed) in activated macrophages [35]. The functions of these genes are unknown but could represent adaptations that distinguish the type II AEC endosome from the macrophage phagosome. “Regulatory proteins” was one of the least represented categories (2%); however, by definition, changes in expression of individual genes in this category can have profound and widespread downstream effects on the expression of other genes. Three genes encoding “Regulatory proteins” were downregulated by *M*. *tb* in this study- Rv3133c (*devR*), Rv31332c (*devS*) and Rv0081. DevR (DosR) and DevS (DosS) are the response regulator and histidine kinase, respectively, of a two-component regulatory system associated with *M*. *tb* survival during a stress-induced, non-replicative, dormant state [36, 37] and Rv0081, a predicted regulatory protein, is itself a member of the DevR-DevS regulon [38]. In contrast, DevR (DosR), DevS (DosS) and Rv0081 as well as many other genes of the DevR (DosR) regulon are upregulated in activated macrophages [35].

**Table 1:** Numbers of *M*. *tb* genes differentially expressed in A549 cells arranged by functional category.

Functional Category^a^	Upregulated	Down-regulated	Total
Conserved Hypotheticals (1042)^b^	18 (1.7%)	28 (2.7%)	46 (4.4%)^c^
Intermediary Metabolism & Respiration (936)	59 (6.3%)	9 (1.0%)	68 (7.3%)
Cell Wall & Cell Processes (772)	38 (4.9%)	14 (1.8%)	52 (6.7%)
Lipid Metabolism (272)	18 (6.6%)	5 (1.8%)	23 (8.5%)
Information Pathways (242)	25 (10.3%)	2 (0.8%)	27 (11.2%)
Virulence, Detoxification, Adaptation (239)	17 (7.1%)	6 (2.5%)	23 (9.6%)
Regulatory Proteins (198)	1 (0.5%)	3 (1.5%)	4 (2.0%)
PE/PPE (168)	4 (2.4%)	2 (1.2%)	6 (3.6%)
Insertion Sequences and Phages (147)	0 (0.0%)	0 (0.0%)	0 (0.0%)
Non-annotated Hypotheticals (N/A)	6 (N/A)	6 (N/A)	12 (N/A)
Total	186	75	261

^a^ Functional categories as designated in the Tuberculist website (http://tuberculist.epfl.ch/index.html).

^b^ Numbers in parentheses below Functional Category indicate total number of genes in the category in the *M*. *tb* H37Rv genome.

^c^ Percentages in parentheses indicate the percentage of genes differentially expressed within the indicated category.

### The DevR (DosR) regulon is downregulated in the intra-AEC environment

The DevR (DosR) *M*. *tb* transcriptional regulator responds to several different stresses believed to exist within the intra-macrophage and/or intra-granuloma environments, e.g., starvation, hypoxia, NO, oxidative stress [36, 38–43]. This regulon consists of at least 48 known genes whose induction is associated with suppressed replication and inhibited aerobic respiration as *M*. *tb* transitions into a persistent state [44]. While the DevR (DosR) response does not drive the pathway to non-replicating “dormancy”, it plays a role in maintaining *M*. *tb* viability during the dormant state [45]. Examination of the differential expression of the DevR (DosR) regulon can provide insight into stresses encountered as well as the respiratory and replicative state of *M*. *tb* in a given environment at a given time.

Twenty six of the 48 DevR (DosR) regulon genes were downregulated by *M*. *tb* during residence in type II AEC; this representation among downregulated genes is statistically significant by Fisher’s Exact test (Table 2); based on fold change these genes were among the most downregulated genes in type II AEC (Table 2 and S1 Table). In contrast, during residence in differentiated human monocytic cell line THP-1 [46] and in IFN-γ activated murine bone marrow derived macrophages [35], 9 and 41 DevR (DosR) regulon genes were upregulated, respectively. These results suggest that the AEC endosome is distinct from the macrophage phagosome (naïve or activated), and may prepare *M*. *tb* for an active rather than a persistent state of infection.

**Table 2:** Differentially expressed functional categories indicative of *M*. *tb* active state in type II AEC.

Functional Group	Description/Association	# of Genes	Hypergeometric Probability (P value)	Range of Fold Change
DevR (DosR) regulon (48)^a^	Dormancy survival	26 (down)	1.529E-24 **(1.583E-24)** ^b^	-2.4 to -44.5
Resuscitation promoting factors (5)	Resuscitation from dormancy	2 (up)	0.03457 **(0.03743)**	+2.0 to +4.2

^a^ Number within parentheses indicates total number of genes in the *M*. *tb* genome within this functional group

^b^ Bold number within parentheses indicates statistical significance, i.e., P value ≤ 0.05

Consistent with this supposition is the upregulation of 2/5 genes encoding “resuscitation promoting factors” (RPFs), Rv1009 (RpfB) and Rv2450c (RpfE), during replication in type II AEC (Table 2); their upregulation constitutes statistical enrichment of this category of genes (Table 2). Deletion of *rpfB* delays *M*. *tb* reactivation in mice [47]. Both RpfB and RpfE interact with RipA which is essential for proper *M*. *tb* cell division [48]. Based on the *in vitro* and *in vivo* phenotypes of sequential *rpf* deletion mutants and single gene complementation of multi-*rpf* mutants, it has been suggested that there is a hierarchy of function among RPFs, with RpfB and RpfE as most influential [49, 50]. Interestingly, no *rpf* genes are differentially expressed by *M*. *tb* in macrophages [35, 46].

### Indicators of *M*. *tb* respiratory state in type II AEC

*M*. *tb* is an obligate aerobe, and utilizes an energy efficient aerobic respiratory pathway during acute infection [51]; however, during exposure to nitric oxide (NO), a product of macrophage activation, this aerobic pathway is inhibited and *M*. *tb* transitions to a non-replicating state [51]. There are two types of NADH dehydrogenase (DH) in *M*. *tb* whose activities contribute to the quinilone pool, NADH DH-1 whose subunits are encoded by genes *nuoA-N* (Rv3145-Rv3158) and two NADH DH-2 encoded by Rv1854c (*ndh*) and Rv0392c (*ndhA*), respectively. NADH DH-1 is utilized during aerobic respiration and the acute stage of infection while NADH DH-2 is utilized during anaerobic electron transfer [51, 52]. When *M*. *tb* is exposed to NO, the NADH DH-2 is used preferentially to drive an alternative electron pathway. Not unexpectedly, 7 (*nuoA*, *B*, *D*, *G*, *H*, *I*, and *J*) of the 14 *M*. *tb* NADH DH-1 subunit genes were upregulated in type II AEC while neither NADH DH-2 encoding gene were differentially expressed (Table 3). This pattern of regulation in type II AEC is statistically significant (Table 3). In contrast, in activated macrophages *M*. *tb* downregulates 6 NADH DH-1 genes and upregulates the NADH DH-2 gene, *ndh* [35].

**Table 3:** Differentially expressed functional categories indicative of *M*. *tb* respiratory and metabolic state in type II AEC.

Functional Group	Description/Association	# of Genes	Hypergeometric Probability (P value)	Range of Fold Change
NADH DH1 (14)^a^	Aerobic respiration	7 (up)	2.66E-05 **(2.90E-05)** ^b^	+2.3 to +5.4
Cytochrome c Reductase (3)	Aerobic respiration	2 (up)	0.01807 **(0.01895)**	+2.5 to +3.6
Cytochrome c Oxidase (4)	Aerobic respiration	1 (up)	0.1888 (0.2076)	+3.2
NADH DH2 (2)	Alternative Electron Transfer	NDE	N/A	N/A
Nitrate Reductase & Transport (5)	Non-aerobic respiration	3 (down)	0.000367 **(0.0003705)**	-2.1 to -6.6

^a^ Number within parentheses indicates total number of genes in the *M*. *tb* genome within this functional group

^b^ Bold number within parentheses indicates statistical significance, i.e., P value ≤ 0.05

Further downstream in the electron flow pathway, during aerobic respiration, cytochrome c reductase (encoded by genes *qrcCAB*) and aa3 cytochrome c oxidase (encoded by genes *ctaCDE*) activities lead to transfer of electrons to oxygen as the final receptor. In contrast, the alternative electron receptor pathway under NO stimulation utilizes nitrate reductase (encoded by genes *narGHJI*) to transfer electrons to nitrate as the final receptor. Consistent with the preferential use of NADH DH-1 in type II AEC, *M*. *tb* also upregulates *qcrA* and *qcrB* of the cytochrome c reductase and *ctaC* of the cytochrome c oxidase and downregulates *narH* and *narI* of the nitrate reductase as well as *narK2* involved in nitrate transport (Table 3). In activated macrophages, *qcrC* and *qcrA* are downregulated as well as *ctaC* and *ctaE* while *narK2* is upregulated [35]. Shi et al., analysed the *M*. *tb* respiratory transcriptome changes that occur over time in the aerosol-infected mouse lung and describe a model of three respiratory states- an acute replicating phase which is evident during the first couple weeks of infection followed by a transitional state lasting through day 50 characterized by inhibition of DH-1 and cytochrome c oxidase, accumulation of nitrate, upregulation of DevR (DosR) and subsequent upregulation of *narK2*, after which *M*. *tb* enters a chronic non-repicating phase [51]. Interestingly, the respiratory transcriptome of *M*. *tb* in type II AEC coincides with the acute replicating phase described by Shi L et al whereas the transcriptome of *M*. *tb* in activated macrophages [35] overlaps with the transitional respiratory state.

### Transcriptional Evidence for Active Replication of *M*. *tb* in Type II AEC

It is well documented that *M*. *tb* invade and replicate in the type II AEC A549 cells [24, 29, 30, 53], and as described above the respiratory transcriptome of *M*. *tb* in these cells correlates with the early acute replicating phase *in vivo*. To further verify a state of active replication, other transcriptional “markers” of replication were evaluated, e.g., indicators of protein synthesis, cell wall synthesis, and a requirement for energy.

The 30S and 50S ribosomal subunits are components of the prokaryotic 70S ribosome which functions as translational machinery in the process of protein synthesis. Of the 23 genes encoding 30S ribosomal proteins, 5 (Rv0682-*rpsL*, Rv2412-*rpsT*, Rv3442c-*rpsI*, Rv3459c-*rpsK*, Rv3460c-*rpsM*) were upregulated in *M*. *tb* replicating in type II AEC (Table 4). In contrast, 13 genes are downregulated in activated macrophages [35]. In addition, 5/36 genes encoding 50S ribosomal proteins (Rv0714-*rplN*, Rv0716-*rplE*, Rv1298-*rpmE*, Rv2441c-*rpmA*, Rv3456c-*rplQ*) were upregulated by *M*. *tb* in type II AEC (Table 4) while 16/36 are downregulated by *M*. *tb* in activated macrophages [35]. The numbers of genes representing each set of bacterial ribosomal proteins upregulated in type II AEC are statistically enriched (Table 4). Furthermore, the gene encoding the elongation factor, Rv0685 (*tuf*) which facilitates translation, was upregulated during replication in type II AEC but was unaffected in the activated macrophage environment (S1 Table; [35]). This pattern of *M*. *tb* differential gene expression in type II AEC is consistent with a demand for increased protein synthesis.

**Table 4:** Differentially expressed functional categories indicative of *M*. *tb* replication in type II AEC.

Functional Group	Description/Association	# of Genes	Hypergeometric Probability (P value)	Range of Fold Change
30S ribosomal proteins (23)^a^	Protein synthesis	5 (up)	0.00677 **(0.008207)** ^b^	+2.0 to +5.6
50S ribosomal proteins (36)	Protein synthesis	5 (up)	0.02796 **(0.03823)**	+2.0 to +3.5
Mycolic acid synthase & FasII enzymes (21)	Cell wall synthesis	7 (up)	0.0001941 **(0.000221)**	+2.8 to +3.7
ATP Synthase (8)	Energy	8 (up)	1.85E-07 **(1.93E-07)**	+2.0- to +5.7

^a^ Number within parentheses indicates total number of genes in the *M*. *tb* genome within this functional group

^b^ Bold number within parentheses indicates statistical significance, i.e., P value ≤ 0.05

Increased bacterial replication necessitates increased synthesis of cell-wall components. Mycolic acids are long chain fatty acids and are among the defining characteristics of the mycobacterial cell wall contributing to “cording” and “acid-fastness” [54, 55]. Genes encoding enzymes involved in mycolic acid synthesis are also statistically represented among *M*. *tb* genes upregulated in type II AEC (Table 4). Among the 21 genes designated as encoding either mycolic acid synthase or FAS II enzymes, 7 were upregulated during replication in type II AEC (Table 4) compared to 3 downregulated in activated macrophages [35].

Finally, active bacterial replication requires energy. All 8 genes that encode subunits of *M*. *tb* ATP synthase were upregulated during infection of type II AEC (Table 4). In stark contrast, during infection of activated macrophages, 7 of the 8 genes are downregulated [35]. Collectively, these transcriptional adaptations to the type II AEC cells are consistent with active *M*. *tb* replication.

### *M*. *tb* Transcriptional Responses to Host Stresses in Type II AEC

*M*. *tb* alters its transcriptional profile in the presence of specific stresses *in vitro*, e.g., hypoxia, starvation, nitric oxide (NO) exposure, acidic pH, etc [39, 56–59]. These stresses and responses are believed to correlate to different environments encountered *in vivo*, within macrophages and granulomas [42, 43]. The heat shock proteins (HSPs) maintain proper folding of proteins, especially under stresses that induce protein misfolding, e.g., increased temperature. Expression of *M*. *tb* HSPs is also induced under stresses like hypoxia and starvation [60]. Of the 10 genes encoding defined HSPs or activators of HSPs in *M*. *tb*, 5 were upregulated while replicating in type II AEC giving statistical significance to this group of differentially expressed genes (Table 5). This is similar to the *M*. *tb* response observed in activated macrophages [35]. While hypoxia likely contributes to *M*. *tb* heat shock response in activated macrophages, the upregulation of HSPs in A549 cells is unlikely to be caused by hypoxia in view of the significant downregulation of hypoxia-induced genes.

**Table 5:** Differentially expressed functional categories indicative of *M*. *tb* response to stress in type II AEC.

Functional Group	Description/Association	# of Genes	Hypergeometric Probability (P value)	Range of Fold Change
Heat shock response (10)^a^	Prevention of protein misfolding	5 (up)	3.83E-04 **(4.15E-04)** ^b^	+2.1 to +5.3
Hypoxia-induced (47)	Inducible by hypoxic conditions ^c^	15 (down)	1.75E-12 **(1.86E-12)**	-2.4 to -44.5
Universal Stress Proteins (9)	Induced during oxygen depletion ^d^	5 (down)	4.69E-06 **(4.83E-06)**	-2.3 to -18.1
Low-pH-induced (23)	Induced by acid shock ^e^	4 (up)	2.62E-02 **(3.30E-02)**	+2.3 to +4.2

^a^ Number within parentheses indicates total number of genes in the *M*. *tb* genome within this functional group

^b^ Bold number within parentheses indicates statistical significance, i.e., P value ≤ 0.05

^c^ (Sherman et al., 2001) [56]

^d^ (O’Toole and Williams, 2004) [61]

^e^ (Fisher et al., 2002) [58]

The majority of DevR (DosR) regulon genes (39/48) are inducible by hypoxic conditions [56]. Sherman et al. identified 47 *M*. *tb* genes that are inducible under hypoxic conditions *in vitro* [56]. Of these, 15 were downregulated during *M*. *tb* replication in type II AEC indicating adequate oxygen availability to *M*. *tb* in these cells (Table 5). This is in contrast to the upregulation of 10 hypoxia-inducible genes in the naïve differentiated human monocytic cell line THP-1 [46] and the upregulation of nearly all (40) in activated murine bone marrow-derived macrophages [35]. Another indication of a paucity of oxygen availability is the universal stress response. Nine genes of the *M*. *tb* genome encode “Universal Stress Proteins” (USPs). These are expressed when oxygen deprivation leads to inhibition of growth [61]. Five of these genes (Rv1996, Rv2028c, Rv2623, Rv2005c, and Rv2026c) were downregulated by *M*. *tb* in type II AEC (Table 5). Again, this is in striking contrast to *M*. *tb* in activated macrophages where *M*. *tb* upregulates 7 USP genes [35]. Genes defined as hypoxia-induced as well as genes encoding USPs exhibit statistical representation among downregulated genes in this study (Table 5). These results suggest that, unlike the intra-phagosomal compartment of activated macrophages, the type II AEC *M*. *tb*-containing endosome is not hypoxic and therefore, hypoxia is not a likely cause for the upregulation of heat shock response genes described earlier. Possibly, the increase in protein synthesis in A549 cells may necessitate an increase in protein folding chaperone activity.

Another potential stress encountered within the bacteria-containing vacuole is low pH as the vacuole matures and acidifies. *M*. *tb* has mechanisms to inhibit the maturation of the macrophage phagosome by reducing acidification and preventing lysosome fusion; however, activation of the macrophages with IFN-γ allows the macrophage to overcome this *M*. *tb* mechanism and acidification can proceed [62]. Phagosomal fusion with lysosomes leads to a pH as low as 4.5. Fisher et al. have identified 23 *M*. *tb* genes that are induced by acid shock, i.e., 15 minute exposure pH 5.5 versus pH 6.9 in 7H9 broth [58]. Four of these genes were upregulated by *M*. *tb* giving statistical significance to this response by the bacteria in type II AEC (Table 5). At 72 hr, *M*. *tb* reside in endocytic vacuoles in A549 [29]. The pH of A549 endosomal compartments has been shown to drop to below 5.3 [63], and the *M*. *tb*-containing compartment in A549 progresses to the late endosomal stage (majority co-localization with Rab7 by 72 hr) [64]. Thus, some overlap between the genes upregulated during acid-shock and in *M*. *tb* residing in type II AEC is expected. During *M*. *tb* infection of macrophages 8/23 acid shock induced genes are upregulated [35, 46].

### Nutrient Status of *M*. *tb* in Type II AEC

The *M*. *tb*-containing macrophage phagosome is known to be nutrient and iron limiting [3, 35]; however, the relative access to these essentials during infection of type II AEC is not defined. None of the genes induced by starvation *in vitro* were upregulated and four (Rv3288c, Rv3287c-*rsbW*, Rv2557, Rv3288c) were downregulated suggesting that *M*. *tb* experiences type II AEC as a nutrient rich environment [39] (S1 Table). Interestingly, many of the *M*. *tb* gene categories described above as upregulated in A549 cells (e.g., genes encoding ATP synthase, NADH DH1, and ribosomal proteins) are downregulated during starvation [39] indicating the type II AEC endosome is a generally nutrient-rich site for *M*. *tb*.

To assess the availability of iron in type II AEC, the expression of three groups of *M*. *tb* genes were examined. Genes involved in the synthesis of siderophores (exochelins, mycobactins), high-affinity iron chelators which capture iron sequestered by host iron-chelating molecules [65], are expected to be upregulated in an iron-limiting environment. Genes within the ESX-3 locus, required for the bacteria to then obtain the iron from the siderophores [66], are also expected to be upregulated in low iron conditions. In an environment where iron is in excess of need, the gene encoding iron storage protein, BfrB, is expected to be upregulated. The pattern of expression of these three groups of genes in *M*. *tb* in type II AEC is consistent with a moderately iron-limited environment. Two of the 14 siderophore biosynthesis genes and 6 of the 11 ESX-3 locus genes were upregulated while *bfrB* was downregulated (Table 6). While the upregulation of 2 siderophore biosynthesis genes does not generate a statistically significant representation, genes of the ESX-3 locus are statistically represented (Table 6). In comparison, *M*. *tb* residing in activated macrophages upregulate 10 siderophore biosynthesis genes indicating lower availability of free iron in the activated macrophage phagosome compared to the type II AEC endosome.

**Table 6:** Differentially expressed functional categories indicative of *M*. *tb* access to iron and nutrients in type II AEC.

Functional Group	Description/Association	# of Genes	Hypergeometric Probability (P value)	Range of Fold Change
Siderophore Biosynthesis (14)^a^	High-affinity iron chelators	2 (up)	1.30E-01 (1.63E-01)^b^	+2.5 to +5.1
ESX-3 Locus (11)	Iron acquisition	6 (up)	9.39E-07 **(9.96E-07)**	+2.0 to +6.3
BfrB (1)	Iron Storage	1 (down)	N/A	-4.0
Tryptophan synthesis (5)	Nutrient; growth factor	3 (up)	4.23E-03 **(4.49E-03)**	+2.2 to +10.1

^a^ Number within parentheses indicates total number of genes in the *M*. *tb* genome within this functional group

^b^ Bold number within parentheses indicates statistical significance, i.e., P value ≤ 0.05

In response to infection, host cells attempt to limit nutrient availability to the intracellular pathogens [67]. Interestingly, three tryptophan synthesis genes, Rv1613 (*trpA*), Rv1612 *(trpB)* and Rv1611 (*trpC*) were upregulated in *M*. *tb* during residence in type II AEC cells (Table 6 and S1 Table) giving statistical significance to the upregulation of this pathway (Table 6). In fact, *trpA* and *trpC* were the two most highly upregulated of all *M*. *tb* genes in the type II AEC environment with 10.1 and 9.5 fold changes, respectively. Indoleamine 2,3-dioxygenase (IDO-1) is a host enzyme that breaks down tryptophan and plays a role in killing of intracellular pathogens [68], and IFN-γ-mediated IDO-1 activity in A549 has been associated with such antimicrobial effects [69]. While IDO-1 is induced in *M*. *tb*-infected human BAL macrophages [70], the effect of *M*. *tb* infection on IDO-1 production in type II AEC is not known. If IDO-1 is also induced in A549 cells during *M*. *tb* infection, the ability of *M*. *tb* to synthesize its own tryptophan may contribute to overcoming the tryptophan limitation in this environment. Interestingly, *M*. *tb* tryptophan auxotrophs are avirulent in mice [71]. These genes are not differentially expressed during infection of naïve or activated macrophages [35, 46].

### Genes Encoding ESAT-6 and ESAT-6-like Proteins are Upregulated During Infection of Type II AEC

While virulent strains of *M*. *tb* are cytotoxic to A549 monolayers and exhibit cell-cell spreading in A549 monolayers, *M*. *bovis* BCG is attenuated for both these phenotypes [26, 28]. *esat-6* transcription was upregulated ~4 fold in *M*. *tb* replicating in type II AEC (S1 Table). The RD1 genomic region which encompasses *esat-6*, and much of the ESX-1 locus which encodes the type VII secretion system required for secretion of ESAT-6 [72, 73] is missing in all BCG strains. *M*. *bovis* BCG, *M*. *tb ΔRD1* and *M*. *tb Δesat-6* mutants are attenuated for dissemination from the lungs of aerosol-infected animals [74–76]. ESAT-6 has been shown to be a laminin-binding adhesin as well as a lysin that binds to A549 cells and causes their lysis [32]. No differential regulation of *esat-6* transcripts is observed in *M*. *tb* during replication in naïve macrophages, and *esat-6* is downregulated in activated macrophages [35, 46]. Together these results suggest that invasion and replication in AEC enables *M*. *tb* to enhance its disseminative potential.

ESAT-6 belongs to a family of 22 additional secreted proteins designated “ESAT-6-like” based on their small size (~100 amino acids), a conserved helix-hairpin-helix structure and a central WXG motif [77]. Genes for 5 ESAT-6-like proteins, Rv0288 (EsxH), Rv1038c (EsxJ), Rv1197 (EsxK), Rv2347c (EsxP), and Rv3620c (EsxW), were also upregulated during *M*. *tb* replication in type II AEC (Table 7), making *esat-6*-like genes statistically represented among upregulated *M*. *tb* genes (Table 7). Of these, *esxH* was the most highly upregulated (>6 fold) (Table 7 and S1 Table). Like *esat-6*, this gene is not upregulated in macrophages [35, 46]. EsxH is encoded in the ESX-3 locus, is specific to the *M*. *tb* complex [78], and is expressed in virulent *M*. *tb* H37Rv but not in the avirulent H37Ra strain [79]. Importantly, EsxH is strongly recognized by PBMCs from TB patients indicating its expression during human infection [80]. Despite low sequence homology to ESAT-6, the structure of EsxH in complex with its binding partner EsxG is highly similar to the structure of ESAT-6/CFP10 complex [81, 82]. The EsxH/EsxG complex contributes to inhibition of phagosome maturation in macrophages [83]; the function of EsxH during infection of type II AEC is not known.

**Table 7:** Genes encoding ESAT-6-like proteins differentially expressed by *M*. *tb* in type II AEC.

Functional Group	Description/Association	# of Genes	Hypergeometric Probability (P value)	Range of Fold Change
Total ESAT-6-like in genome (23)^a^	Similar structure to ESAT-6	6 (up)	1.52E-03 **(1.79E-03)** ^b^	+2.1 to +6.3
ESAT-6-like Outside ESX loci (12)	Functions unknown	4 (up)	4.56E-03 **(5.13E-03)**	+2.1 to +3.0

^a^ Number within parentheses indicates total number of genes in the *M*. *tb* genome within this functional group

^b^ Bold number within parentheses indicates statistical significance, i.e., P value ≤ 0.05

The other 4 upregulated ESAT-6-like family genes, *esxJ*, *esxK*, esx*P*, and *esxW*, lie outside of any ESX loci (Table 7). Interestingly, although these 4 proteins also have little sequence similarity to ESAT-6, they have ~98% identity amongst themselves and belong to a small sub-family of the ESAT-6-like family characterized by a C-terminal “QILSS” motif [84]. The gene products also belong to the “Mtb9.9 family” of secreted T cell antigens [80, 85, 86]. In the avirulent *M*. *tb* H37Ra, the four corresponding genes have multiple mutations including stop codons in *esxP* and *esxW* [87]. *esxP* and *esxW* are located within RD5 and RD8, respectively, regions present in *M*. *tb* but missing from most *M*. *bovis* BCG strains [84]. Interestingly, *esxK* and *esxP* are highly expressed in sputum from patients with active TB [88], and *M*. *tb*-infected AEC have been reported to be present in sputum [88]. The structures of these proteins have not been solved; however, *in silico* modeling of EsxW in complex with Rv3619c (EsxV) shows remarkable similarity to the ESAT-6:CFP-10 complex and the EsxH:EsxG complex [81, 82, 89]. None of these genes (*esxJ*,*K*,*P*,*W*) are differentially expressed during infection of THP-1 or activated mBMM [35, 46]. These results suggest that EsxJ, EsxK, EsxP and EsxW have as yet unknown function(s), which may be important to *M*. *tb* during replication in type II AEC and subsequent dissemination from the lungs.

### Upregulation of Genes Encoding Adhesins and Other Potential Host Interaction Proteins

Adhesins are extracellular or surface exposed proteins that promote bacterial interactions with the host via binding to specific extracellular matrix proteins (ECM). ESAT-6 binds AEC and specifically to laminin, an ECM protein found both on AEC membranes and as the predominant component of the basement membrane (BM) underlying AEC [32]. We have postulated that ESAT-6 may contribute to *M*. *tb* dissemination from the lung by exposing the BM through lysis of AEC and anchoring *M*. *tb* to the BM via its interaction with laminin [32]. Whether EsxH or the other ESAT-6-like proteins described above have similar properties has yet to be investigated. Another *M*. *tb* adhesin, Rv1837c (glcB, malate synthase- MS) was also upregulated in *M*. *tb* replicating in type II AEC (2.88 fold) (S1 Table). *M*. *tb* MS has been shown to bind laminin and fibronectin, and to type II AEC in a laminin-dependent manner [33]. Rv1566c and Rv2190c, also upregulated in this study (5.22 fold and 3.90 fold, respectively) (S1 Table) have domains that belong to the NLPC_p60 family found in bacterial proteins involved in host cell invasion [90–92]. Also upregulated in A549 cells was Rv0129c (4.83 fold) (S1 Table) which encodes, Ag85C, one of three secreted fibronectin-binding and elastin-binding proteins of the immunogenic Ag85 complex [93–95]. Rv3922c, upregulated (2.79 fold) (S1 Table), encodes a protein of unknown function in *M*. *tb*; however, due to its high similarity to the *Aeromonas hydrophila* α-hemolysin, it is described as a possible hemolysin (tuberculist.epfl.ch). Upregulation of Rv1566c, Rv2190c, Rv0129c, and Rv3922c in type II AEC could contribute to the *M*. *tb*/host interaction. Interestingly, the recently described *M*. *tb* adhesin, Rv0931c (PknD), which is a secreted, laminin-binding protein that contributes to infection of the human brain microvascular endothelial cell (but not macrophages, AEC, or other endothelial cells) and to invasion of the blood-brain barrier by *M*. *tb* animal studies [96] was not upregulated by *M*. *tb* replicating in type II AEC. The absence of differential expression of *pknD* in type II AEC, suggests cellular specificity with regards to *M*. *tb* adhesin expression.

### Verification of Select Genes by quantitative RT-PCR

To verify the differential expression of a few of these genes, unamplified RNA from *M*. *tb* H37Rv replicating in A549 (the same conditions as for microarray analysis) was analyzed by qRT-PCR as was RNA extracted from *M*. *tb* H37Rv grown logarithmically in laboratory broth (Table 8 and S1 Table). As expected, transcripts of the 23S rRNA (negative control) did not show differential expression by qRT-PCR. Transcripts for *esat-6* were upregulated ~3.8 fold by microarray and ~4.8 fold by qRT-PCR. Similarly transcripts for *esxH* were upregulated ~6.3 fold by microarray and ~10.0 fold by qRT-PCR. Thus, in both assays *esxH* was more highly upregulated than *esat-6*. Among genes identified as downregulated by microarray, genes of the DevR (DosR) regulon were statistically enriched (Table 2); many of these genes have been found to be upregulated by *M*. *tb* in activated macrophages [35]. Two of these genes, Rv3132c (*devS*, *dosS*) and Rv3131, were tested by qRT-PCR for verifying their downregulation during infection of type II AEC. Specifically, Rv3132c (devS, *dosS*) was found to be down ~7.5 fold by microarray and ~2.4 fold by qRT-PCR. Rv3131, the most downregulated gene (~44 fold) observed by microarray, was found to be tremendously downregulated by qRT-PCR (10 fold greater than by microarray). The fold changes for each of the above described genes are statistically different from the negative control, 23S (Table 8). *pknD* was not differentially expressed by microarray, and there was no significant difference between the transcripts for PknD and the 23S negative control by qRT-PCR.

**Table 8:** quantitative RT-PCR verification of select genes.

Differential Expression (by microarray)	*M*. *tb* Gene	Fold Change^a^ (+/- SD) (by qRT-PCR)	P-value *
Upregulated	Rv3875 (*esat-6*)	4.84 (+/- 1.87)	**0.0218**
	Rv0288 (*esxH*)	10.04 (+/- 0.79)	**<0.0001**
Downregulated	Rv3132c (*devS*)	0.4132 (+/- 0.0650)	**0.0047**
	Rv3131	0.002009 (+/- 2.36E-04)	**0.0003**
Not Differentially Expressed	Rv0931c (*pknD*)	2.23 (+/- 1.20)	0.1269
Negative Control	23S rRNA	0.89 (+/- 0.13)	N/A

^a^Fold change indicates the ratio of the normalized number of transcripts for the select gene during *M*. *tb* infection of A549 cells to the normalized number of transcripts of the same gene during mid-log growth of *M*. *tb* in 7H9 media with standard deviation in parentheses.

*P-value indicates the statistical comparison of fold change of the select gene to fold change of the negative control (23S RNA) by unpaired two-tail t test. P-values ≤ 0.5 are statistically significant and are highlighted in bold.

## Concluding Remarks

Our inability to precisely identify the time of infection with *M*. *tb* and the subsequent lack of any clinical indications makes it impossible to delineate the interaction that occurs during the first host-pathogen encounter post-inhalation resulting in systemic establishment of infection in humans. However, studies in animal models indicate dramatic bacterial replication in a non-migrating, non-antigen-presenting compartment in the lungs and bacterial migration from the lungs to multiple organs prior to elicitation of cellular immune responses [9–11]. Together, these studies implicate the AEC as an important niche for *M*. *tb* replication during primary infection and as a probable route for dissemination.

The intra-type II AEC *M*. *tb* transcriptome defined in this study is consistent with the active bacterial replication reported post-infection in animal models [11] and to studies performed with the AEC cell line A549 [31]. Thus, multiple genes that are involved in protein synthesis (e.g., genes encoding 30S and 50S ribosomal subunits), cell-wall synthesis (e.g., genes involved in mycolic acid biosynthesis) and energy production (e.g., genes encoding ATP synthase subunits) were upregulated (Table 4), while genes associated with dormant, non-replicating bacteria (e.g., DevR (DosR) regulon) were downregulated (Table 2), as were genes typically induced under stresses that lead to suppressed replication such as hypoxia and starvation (Table 5).

Multiple *in vivo* studies have demonstrated the requirement for ESAT-6 expression and secretion for virulence and dissemination of *M*. *tb* [74–76]. Studies with A549 cells have also demonstrated that virulent strains of *M*. *tb* lyse A549 cells and spread in A549 monolayers [23, 26, 28]. Further, strains that do not express or secrete ESAT-6 are attenuated both for dissemination *in vivo* and lysis of A549 cells and spreading in A549 monolayers *in vitro* [23, 26, 28, 75]. The strong upregulation of *esat-6* transcripts (~4 fold) during *M*. *tb* replication in A549 cells correlates with these earlier results. Interestingly, lack of ESAT-6 activity attenuates, but does not abrogate, the dissemination of *M*. *tb*, indicating the potential involvement of additional factors. While the role of gene products of *esxJ*, *esxK*, *esxP*, *and esxW* are as yet unknown, the predicted secondary and tertiary structures suggest the potential for similar functions. Interestingly, in addition to the predominant upregulation of *esat-6*, transcripts for *esxJ*, *esxK*, *esxP*, *and esxW* were upregulated in *M*. *tb* replicating in blood from HIV+ patients but not in blood from HIV- donors [97]. Since TB frequently manifests as disseminated, miliary TB and/or extrapulmonary TB in persons co-infected with HIV, the correlation of upregulation of their transcripts with *in vivo* environments where dissemination is likely occurring also suggests that these proteins may contribute to bacterial dissemination [98–100].

Other Gram-positive pathogens such as *Streptococci*, *Staphylococci*, *Listeria* etc use the coordinated activities of repertoires of adhesins and lysins to translocate across mucosal barriers [101–109]. Thus, *S*. *pneumoniae* (*S*. *pn*) use CbpA (choline binding protein A; adhesin), PspA (pneumococcal surface protein A; adhesin) and Ply (pneumolysin; lysin) for infection and dissemination from the lungs [103, 110]. Double mutants, *Δply pspA S*.*pn* and *Δply cbpA S*. *pn*, are more attenuated than single mutants demonstrating their co-operative function *in vivo* [111, 112]. Similarly, for causing pneumonia, *S*. *aureus* uses FnBP (Fn binding protein) for colonization and adhesion to the respiratory epithelium and α-hemolysin to damage the alveolar barrier to promote hematogenous dissemination of the bacteria [101, 113]. Besides *esat-6* and genes encoding ESAT-6-like proteins, transcripts for other known and potential adhesins and lysins of *M*. *tb* were also upregulated during *M*. *tb* replication in AEC. These include the known adhesin, GlcB/malate synthase (Rv1837c), described previously by our lab [33], the fibronectin-binding and elastin-binding Ag85C, and the possible lysin, Rv3922c. Also upregulated were transcripts for Rv1566c and Rv2190c, which encode proteins with NLPC_p60 domains; the presence of these domains is associated with other mycobacterial proteins important for invasion of macrophages [92]. This suggests that Rv1566c and Rv2190c could potentially contribute to invasion of cells of the alveolar barrier. The coordinated actions of one or more of these known or potential adhesins and lysins in *M*. *tb* dissemination need further investigation.

There are three outcomes from *M*. *tb* exposure- clearance, establishment of latent infection, or progression to active disease. While it is believed that the alveolar macrophages host *M*. *tb* during primary infection, and the mechanisms used by *M*. *tb* to survive and replicate within these bacteriacidal cells have been studied extensively, these are not the only cells the inhaled bacteria encounter during primary infection. Other innate immune cells, including neutrophils, natural killer cells, mucosal associated invariant T cells and γδ T cells could also contribute to the early clearance of *M*. *tb* [114]. The AECs greatly outnumber macrophages and the other cells that confer innate immunity in the alveolus. The *M*. *tb* transcriptome in type II AEC indicates an aerobic, actively replicative state, while the transcriptome in the intra-macrophage environment (especially the intra-“activated” macrophage environment) resembles transition to dormancy [51]. This leads to the hypothesis that while uptake of *M*. *tb* by macrophages may favor transition to dormancy, invasion of and replication in type II AECs during primary infection may serve to avoid early clearance and favor not only establishment of systemic infection but also progression to active disease.

Since the current vaccine, the live attenuated *M*. *bovis* BCG, is highly variable for protection against pulmonary TB in adults [115], and the most recent vaccine trial of MVA85A, a viral-vector carrying *M*. *tb* antigen 85A, was completely unsuccessful in boosting protection by BCG [116], new strategies in anti-TB vaccine development are urgently needed. This quest is greatly hampered by the fact that correlates of protection against *M*. *tb* infection in humans are poorly understood. The majority of current TB vaccine development is based on the induction of Th1 cellular immune responses in animal models of TB [117]; however, it is clear that this is not sufficient to predict protection in humans. In the current study, we have described the transcriptional profile of *M*. *tb* within type II AEC as an active and disseminative state of infection. The type II AEC, therefore, is a potential niche, allowing *M*. *tb* to replicate to large numbers and spread systemically while simultaneously evading the innate immune mechanisms responsible for early clearance. Targeting *M*. *tb* interaction with and/or survival within type II AECs may be a novel and complementary strategy for preventing infection or progression to active disease particularly in those known to have been exposed to *M*. *tb*.

## Materials and Methods

### A549 culture and infection with *M*. *tb*

A549 cells (ATCC; CCL-185) were cultured in 37°C 5% CO_2_ in RPMI supplemented with 7.5% FBS, 2 mM L-glutamine, 1% MEM nonessential amino acids in ~5–7 x 225cm^2^ flasks (Corning) per experiment. Once confluent, cells of one flask were detached with RPMI/ 500 mM EDTA for counting. This number was used to determine a bacterial inoculum consistent with an MOI of ~5:1 (5 bacteria/cell). *M*. *tb* H37Rv was grown to mid-log phase in Middlebrook 7H9 broth as previously described [97], and single cell suspensions used to infect A549 monolayers in the remaining flasks for 1 hr. The infected cells were washed extensively with warm RPMI to remove extracellular bacteria and maintained in the supplemented RPMI containing 1% FBS, 2 mM L-glutamine and 1% non-essential amino acid for 3 days at 37°C 5% CO_2_.

### Isolation of *M*. *tb* from A549 and extraction of *M*. *tb* RNA

At 3 days pi, infected cells were washed twice with warm RPMI then lysed with GTC solution (4 M guanidinium thiocynate, 0.5% sodium N-lauryl sarcosine, 25 mM trisodium citrate, 0.1 M 2-mercaptoethanol, 0.5% Tween 80, pH 7.0) and centrifuged at 5000 *g* for 20 min at 4°C to pellet bacteria [118]. The pellets were washed in GTC solution and resuspended in TRI reagent containing polyacrylamide carrier (Molecular Research Center, Cincinnati, OH, USA). Total RNA was extracted using the protocol described previously [46]. Reference RNA from *M*. tb H37Rv grown to log phase in 7H9 broth was extracted similarly. The reference bacteria was cultured as follows. Briefly, 10 ml Middlebrook 7H9 media was inoculated with a 0.5 ml frozen aliquot of bacteria (OD600 = 0.7 at time of storage) yielding a starting culture of OD600 = 0.035. The culture was incubated at 37°C with shaking (110 rpm) for 7–9 days and harvested at an OD600 of 0.7–0.9 (mid to late log phase) [97].

### DNA Microarrays

RNA from *M*. *tb* cultured in A549 cells for 3 days from two experiments (two biological replicates) was used for the DNA microarray studies with RNA from *M*. *tb* grown to log phase in Middlebrook 7H9 broth as the reference. RNA quality and quantity were verified by Agilent Bioanalyzer 2100, and RNA integrity numbers for each experimental RNA sample F1, F2, and 2F, were 8.7, 8.6, and 7.7, respectively, and 9.5 for reference RNA. To ensure adequate amounts of RNA for the microarray study, an RNA amplification strategy was used on similar amounts of all RNA samples from *M*. *tb* grown in A549 and Middlebrook 7H9 [97, 119, 120]. This strategy was verified by microarray previously in our laboratory to confirm that the expression profiles of amplified *M*. *tb* RNA (aRNA) are similar to those of unamplified RNA [97]. The *M*. *tb* microarray chips containing 70-mer oligonucleotides representing all open reading frames annotated in the *M*. *tb* H37Rv genome sequence were obtained from the Center for Applied Genomics (Public Health Research Institute; Newark, NJ). Labeled cDNA from aRNA of log phase *M*. *tb* grown in 7H9 broth was used as the reference control on each chip. For hybridization, labeled cDNA probes generated from aRNA obtained from *M*. *tb* grown in each A549-grown biological replicate was mixed with the labeled reference cDNA probe prior to purification with Microcon YM10 filter (Millipore). The *M*. *tb* arrays were hybridized overnight with the mixed labeled cDNA probes in duplicate to include a Cy3/Cy5 dye flip [46]. For one biological sample, four technical replicates were used (including two dye flips) and for the other biological sample, two technical replicates were used (one dye flip). The microarrays were scanned and processed with an Axon 4000B scanner and GenePix Pro 6.1 software, respectively. The chips were normalized by the print-tip Lowess method, and the Cy5/Cy3 intensity ratio was determined for each gene [121]. The intensity ratio data obtained from *M*. *tb* microarray chips was used to perform Significance Analysis of Microarrays (SAM) with Multiarray Viewer Software on the TMEV website for determination of differentially expressed genes of *M*. *tb* grown in A549 cells compared to 7H9 broth grown *M*. *tb* [122]. Among genes identified by SAM, only genes that showed a ≥ 2 fold change at a false discovery rate of <2% were considered significantly differentially expressed. Data were deposited in Gene Expression Omnibus repository (Accession Number: GSE58466). The general functional categorization for *M*. *tb* differentially expressed genes was determined as described on the TubercuList website (http://tuberculist.epfl.ch/).

### Statistical Analysis

The heat map was generated using TMEV MeV_4_9_0 software. Fisher's Exact Test (Microsoft Fisher's Exact Test Calculator) was applied to the data to determine the hypergeometric probability of representation of specific functional gene categories among differentially expressed genes.

### Quantitative RT-PCR

Expression of selected genes was determined in unamplified RNA from *M*. *tb* grown in A549 and from *M*. *tb* grown logarithmically in 7H9 Middlebrook laboratory broth by qRT-PCR [32]. *M*. *tb* gene specific primers were designed using Primer3 software (S2 Table). Briefly, *M*. *tb* RNA was subjected to synthesis of first-strand cDNA using Superscript II RNase H^-^ reverse transcriptase (RT). The real-time PCR was performed using iQ SYBR Green supermix, primers, and cDNA in a MyiQ2 two color Real Time PCR Detection System (Bio-Rad). For each RNA sample, a reaction without RT was performed as a negative control. For quantitation, a standard curve was generated for each gene using a serial dilution of *M*. *tb* genomic DNA and respective primers. Copies of 16S rRNA was used to normalize the transcript levels of the respective genes. The ratio of normalized copies of each selected gene from *M*. *tb* grown in A549 to normalized copies in 7H9 broth-grown *M*. *tb* was calculated to determine the fold change in expression of that gene in A549 compared to reference 7H9 broth. Prior to quantitation, the specificities of PCR products were verified by amplification of each gene target using respective primers with *M*. *tb* H37Rv genomic DNA as templates and sequencing of amplified products. Unpaired two-tailed t test was applied to determine statistical differences between fold changes of selected genes and the fold change of 23S (negative control) using GraphPad Prism 6 soft ware.

## Supporting Information

S1 Table Complete Listing of *M*. *tb* Genes Upregulated and Downregulated at 72 hr Replication in A549. (PDF)

S2 Table Sequences of Primers Used in qRT-PCR. (PDF)
