# Supplementary material for: Transcriptional Profile of Mycobacterium tuberculosis Replicating in Type II Alveolar Epithelial Cells
Source: PLoS One. 2015 Apr 6;10(4):e0123745. doi: 10.1371/journal.pone.0123745 (PMC4386821; doi:10.1371/journal.pone.0123745)
Supplement: S1 Table — (PDF) [file pone.0123745.s001.pdf]

S1 Table. Complete Listing of M. tb Genes Upregulated and Downregulated at 72 hr Replication in A549.

Upregulated			Downregulated		
Name	ID	Average Fold Change	Name	ID	Average Fold Change
trpA	Rv1613	10.13874141	Rv3131	Rv3131	-44.4702
trpC	Rv1611	9.495824906	Rv1813c	Rv1813c	-27.5369
lgt	Rv1614	7.753575046	Rv3127	Rv3127	-20.3109
nadC	Rv1596	7.452046449	Rv3129	Rv3129	-20.1236
aceA	Rv0467	7.231659079	Rv1996	Rv1996	-18.1025
Rv3281	Rv3281	6.647422259	Rv2028c	Rv2028c	-15.3132
Rv0288	Rv0288	6.274889267	Rv3130c	Rv3130c	-13.165
atpE	Rv1305	5.74001033	Rv3128c	Rv3128c	-12.5056
rpsM	Rv3460c	5.624831972	Rv2629	Rv2629	-11.1347
nuoH	Rv3152	5.437361193	Rv2004c	Rv2004c	-9.73101
Rv0462	Rv0462	5.368007424	Rv1735c	Rv1735c	-9.69429
nuoJ	Rv3154	5.35291595	Rv2631	Rv2631	-8.29898
Rv0430	Rv0430	5.332683358	Rv2623	Rv2623	-8.25507
groES	Rv3418c	5.288700704	Rv2005c	Rv2005c	-8.08869
Rv1566c	Rv1566c	5.22434438	Rv1998c	Rv1998c	-7.70666
trpE2	Rv2386c	5.090257596	Rv3132c	Rv3132c	-7.50482
Rv1751	Rv1751	5.055638588	Rv2630	Rv2630	-6.92652
lpqU	Rv1022	4.966071386	Rv1733c	Rv1733c	-6.86095
fbpC2	Rv0129c	4.829040847	hypothetic	ORF00778	-6.73024
conserved	ORF0175(	4.798610339	narK2	Rv1737c	-6.55151
conserved	ORF01755	4.681848767	Rv3485c	Rv3485c	-6.48297
Rv2199c	Rv2199c	4.680453535	Rv3133c	Rv3133c	-6.46767
fdxC	Rv1177	4.563376433	hypothetic	ORF03834	-6.39573
dnaE1	Rv1547	4.540312714	hypothetic	ORFD0232	-5.8829
pra	Rv1078	4.445841966	Rv2627c	Rv2627c	-5.7431
dnaJ	Rv0352	4.353809649	hspX	Rv2031c	-5.53769
rnpA	Rv3923c	4.270125091	Rv3662c	Rv3662c	-5.3617
Rv2450c	Rv2450c	4.195569055	PPE	Rv3125c	-5.01596
atpF	Rv1306	4.161888554	lipF	Rv3487c	-4.80883
PE	Rv0285	4.136746829	PE_PGRS	Rv2396	-4.16828
ppiA	Rv0009	4.028915832	bfrB	Rv3841	-3.95024
infA	Rv3462c	3.976810084	Rv3197	Rv3197	-3.80863
Rv2190c	Rv2190c	3.902170096	hypothetic	ORF01250	-3.80231
nuoI	Rv3153	3.895375755	Rv3371	Rv3371	-3.73713
excisionas	ORF0504(	3.854352003	Rv2876	Rv2876	-3.52546
dnaK	Rv0350	3.821877843	Rv2033c	Rv2033c	-3.50929
esat6	Rv3875	3.779346165	ctpF	Rv1997	-3.49806
acpM	Rv2244	3.745976027	Rv3081	Rv3081	-3.35131
hupB	Rv2986c	3.696402005	Rv0736	Rv0736	-3.34846
adk	Rv0733	3.686746363	Rv2625c	Rv2625c	-3.29567
inhA	Rv1484	3.659398846	Rv2867c	Rv2867c	-3.18883
qcrA	Rv2195	3.637242209	Rv1999c	Rv1999c	-3.07677

Rv1591	Rv1591	3.60218098
rpsK	Rv3459c	3.595207402
kasA	Rv2245	3.539506357
atpH	Rv1307	3.529184858
rplI	Rv0056	3.493375358
glgP	Rv1328	3.417401684
ansP	Rv2127	3.402035825
pstC	Rv0935	3.296130433
tuf	Rv0685	3.294728685
Rv2951c	Rv2951c	3.289524368
sodA	Rv3846	3.237539587
Rv0466	Rv0466	3.212946222
Rv0983	Rv0983	3.208276166
ctaC	Rv2200c	3.200692323
bioB	Rv1589	3.168334886
lprO	Rv0179c	3.140332891
Rv2817c	Rv2817c	3.121998477
Rv1770	Rv1770	3.116238528
Rv0636	Rv0636	3.088497151
Rv3620c	Rv3620c	3.043701053
rpmH	Rv3924c	3.011667153
atpB	Rv1304	3.006697373
Rv3200c	Rv3200c	3.002731895
metZ	Rv0391	2.990693367
Rv3408	Rv3408	2.990050809
Rv0284	Rv0284	2.965270738
rpmE	Rv1298	2.942861202
Rv3075c	Rv3075c	2.938610537
gyrB	Rv0005	2.937998515
PPE	Rv0286	2.92804285
Rv1747	Rv1747	2.878690792
glcB	Rv1837c	2.875922569
umaA1	Rv0469	2.859025194
metB	Rv1079	2.847808561
echA7	Rv0971c	2.840350232
Rv0637	Rv0637	2.820271069
nuoA	Rv3145	2.809063038
Rv3922c	Rv3922c	2.792127844
whiB1	Rv3219	2.724236902
atpD	Rv1310	2.718690019
Rv0463	Rv0463	2.691305191
ponA	Rv0050	2.68686096
secD	Rv2587c	2.686053735
tpi	Rv1438	2.676832438
Rv0461	Rv0461	2.622439215
PE_PGRS	Rv2162c	2.611387404
Rv1303	Rv1303	2.607385078

Rv2003c	Rv2003c	-3.01515
nirA	Rv2391	-3.00631
narH	Rv1162	-2.97158
Rv0081	Rv0081	-2.79979
Rv1535	Rv1535	-2.78924
Rv2557	Rv2557	-2.67046
Hypotheti	ORF08416	-2.63071
Rv3108	Rv3108	-2.59654
Rv1682	Rv1682	-2.58456
Rv1639c	Rv1639c	-2.53071
Rv1739c	Rv1739c	-2.49415
Rv2777c	Rv2777c	-2.42411
glnD	Rv2918c	-2.42056
Rv0079	Rv0079	-2.41027
Rv1184c	Rv1184c	-2.38241
Rv2136c	Rv2136c	-2.36747
rsbW	Rv3287c	-2.34758
Rv2026c	Rv2026c	-2.31156
Rv3288c	Rv3288c	-2.29767
papA5	Rv2939	-2.28041
Rv2516c	Rv2516c	-2.25427
pks17	Rv1663	-2.24526
Rv2694c	Rv2694c	-2.17356
Rv2076c	Rv2076c	-2.17178
Rv0459	Rv0459	-2.16322
narI	Rv1164	-2.13642
Rv3848	Rv3848	-2.07718
rfe	Rv1302	-2.06321
Rv1772	Rv1772	-2.03988
pks3	Rv1180	-2.03859
Rv0919	Rv0919	-2.02524
conserved	ORF00615	-1.99894
Rv2406c	Rv2406c	-1.98743

Rv1700	Rv1700	2.598092267
Rv3716c	Rv3716c	2.59207956
pheT	Rv1650	2.590677022
ssb	Rv0054	2.580107482
nadB	Rv1595	2.571553794
Rv1544	Rv1544	2.567282399
ruvC	Rv2594c	2.555782382
Rv2603c	Rv2603c	2.525763106
nuoK	Rv3155	2.51027427
Rv0049	Rv0049	2.500365497
qcrB	Rv2196	2.495956573
map'	Rv0734	2.489259553
nuoD	Rv3148	2.459263519
mbtH	Rv2377c	2.452736922
hns	Rv3852	2.429646626
ahpD	Rv2429	2.424248067
metE	Rv1133c	2.417236
ftsX	Rv3101c	2.416717767
rpmA	Rv2441c	2.401594647
nuoG	Rv3151	2.398448563
PPE	Rv0442c	2.392646341
atpC	Rv1311	2.378761009
pykA	Rv1617	2.358943722
Rv0247c	Rv0247c	2.358318108
Rv1211	Rv1211	2.353382516
echA3	Rv0632c	2.338171631
Rv2959c	Rv2959c	2.323416021
Rv0892	Rv0892	2.316020195
Rv3747	Rv3747	2.305946966
Rv0954	Rv0954	2.305104518
htpG	Rv2299c	2.299760301
nuoB	Rv3146	2.279498827
kasB	Rv2246	2.274519547
atpG	Rv1309	2.271108556
ahpC	Rv2428	2.256787635
Rv0517	Rv0517	2.254954776
Rv0248c	Rv0248c	2.246566862
Rv0460	Rv0460	2.244446436
fum	Rv1098c	2.229742843
Rv0290	Rv0290	2.224275512
Rv2454c	Rv2454c	2.214909113
rhlE	Rv3211	2.191127091
rpsI	Rv3442c	2.181750055
Rv3199c	Rv3199c	2.174661469
Rv1456c	Rv1456c	2.174167362
Rv2096c	Rv2096c	2.174053083
cspA	Rv3648c	2.172667393

trpB	Rv1612	2.169747006
Rv0289	Rv0289	2.1689691
fadA6	Rv3556c	2.161376145
lprB	Rv1274	2.160610974
Rv3748	Rv3748	2.147204599
rplN	Rv0714	2.145102124
Rv1233c	Rv1233c	2.143149265
fadD28	Rv2941	2.132105263
Rv1197	Rv1197	2.128736184
hypothetica	ORF00414	2.128582489
Rv1478	Rv1478	2.121466651
fadD26	Rv2930	2.120199468
Rv1241	Rv1241	2.118684352
Rv1435c	Rv1435c	2.114811876
adhC	Rv3045	2.110927212
Rv0282	Rv0282	2.109290058
Rv3723	Rv3723	2.108042812
Rv1038c	Rv1038c	2.107813418
Rv2347c	Rv2347c	2.101092727
rpsL	Rv0682	2.098713159
tesA	Rv2928	2.087355989
rubB	Rv3250c	2.075512806
Rv1508c	Rv1508c	2.071844652
cysS	Rv3580c	2.069517813
pgk	Rv1437	2.065970279
Rv2602	Rv2602	2.059181028
groEL2	Rv0440	2.052062967
rplE	Rv0716	2.051659524
Rv2376c	Rv2376c	2.050870646
pgsA	Rv2612c	2.035569999
Rv1713	Rv1713	2.022458127
fadD31	Rv1925	2.021256855
ribosomal	ORF05284	2.020387471
Rv0292	Rv0292	2.020339156
Rv1114	Rv1114	2.017581101
Rv0760c	Rv0760c	2.015847031
Rv1827	Rv1827	2.010631925
ccsA	Rv0527	2.005931953
Rv1926c	Rv1926c	2.003717796
conserved	ORF03012	2.002042823
Rv1778c	Rv1778c	2.000707366
rpsT	Rv2412	1.988154904
gltA2	Rv0896	1.980259502
lpqE	Rv3584	1.978755463
gnd2	Rv1122	1.972404797
rplQ	Rv3456c	1.97044753
Rv1009	Rv1009	1.964895836

pyrH	Rv2883c	1.957008244
Rv3222c	Rv3222c	1.956625298
atpA	Rv1308	1.951328944
