# Supplementary material for: Transcriptional Profile of Mycobacterium tuberculosis Replicating in Type II Alveolar Epithelial Cells
Source: PLoS One. 2015 Apr 6;10(4):e0123745. doi: 10.1371/journal.pone.0123745 (PMC4386821; doi:10.1371/journal.pone.0123745)
Supplement: S2 Table — (PDF) [file pone.0123745.s002.pdf]

S2 Table. Sequences of Primers Used in qRT-PCR.

***M. tb* gene**	**Primer**	**Sequence**
Rv3875 (*esat-6, esxA*)	Forward	5’ TCCATTCATTCCCTCCTTGA 3’
	Reverse	5’ TTTGCTTGGACACCCTGGTA 3’
Rv0288 (*esxH*)	Forward	5’ ATACCGGGATCACGTATCAG 3’
	Reverse	5’ CATGGTGTTGGCTTCATGG 3’
Rv3132c (*devS, dosS*)	Forward	5’ AGCATCGCAGGGTATCACTC 3’
	Reverse	5’ ATGGACCCACGAATTGAAC 3’
Rv3131	Forward	5’ ATGAACACCCATTTCCCGGA 3’
	Reverse	5’ AGAACAGCTCCAGACTCGTC 3’
Rv0931c (*pknD*)	Forward	5’ ATCCATGACTACGGCGAGAT 3’
	Reverse	5’ GGTCAGCGGACCATACTGTT 3’
23S rRNA	Forward	5’ GTGAGCGACGGATTCGCCTAT 3’
	Reverse	5’ ACCACCCAAAACCGGATCGAT 3’
16S rRNA	Forward	5’ GTGGCGAACGGGTGAGTAAC 3’
	Reverse	5’ ARGCATCCCGTGGTCCTATC 3’
